# Children’s gut microbiota predicts the efficacy of obesity treatment

**DOI:** 10.1080/19490976.2026.2631824

**Published:** 2026-02-19

**Authors:** Mireia Alcázar, Verónica Luque, Natalia Ferré, Judit Muñoz-Hernando, Mariona Gispert-Llauradó, Ricardo Closa-Monasterolo, Albert Feliu, Gemma Castillejo, Joaquín Escribano

**Affiliations:** a Paediatric Nutrition and Human Development Research Unit, Universitat Rovira i Virgili, C/Sant Llorenç, 21, Reus, Spain; b Institut de Recerca Biomèdica CatSud, Reus, Spain; c Hospital Universitari Sant Joan de Reus, Av Dr Josep Laporte, 2, Reus, Spain

**Keywords:** Obesity, children, gut microbiota, intervention, weight loss

## Abstract

**Background & Objective:**

Responses to dietary interventions may vary depending on baseline gut microbiota composition. This study aimed to determine whether baseline gut microbiota diversity and composition predict the effectiveness of childhood obesity interventions.

**Methods:**

Anthropometry, triglycerides, HDL-cholesterol, HOMA-IR, and systolic and diastolic blood pressure (SBP, DBP) were evaluated and standardised in 41 children with obesity (8–14yrs). Faecal samples were collected at baseline and after one year. Intervention success was defined by improvements in metabolic risk score (MetScore) or BMI z-score. Associations between baseline microbiota features (diversity and composition) and intervention success were evaluated using Spearman’s correlation and linear regression models. Gut microbiota composition and differential abundance were analyzed using ANCOM-BC2. Exploratory biomarker discovery was analyzed using LEfSe, and predictive modelling using a Random Forest (RF) classifier. Receiver operating characteristic (ROC) curve analysis was used to determine a Simpson index cut-off.

**Results:**

Higher baseline Shannon and Simpson indices, and greater abundances of *Faecalibacterium* and *Eubacterium coprostanoligenes* group, were associated with greater improvements in MetScore. *Faecalibacterium* was the most influential feature with the highest importance in the RF model, which achieved an AUC of 0.876 for MetScore and 0.873 for BMI z-score improvement. Eighty-four features differed between MetScore response groups (FDR < 0.05) with some genus-level overlap with the exploratory analysis, including *Eubacterium coprostanoligenes* and *Ruminococcus*. A Simpson index cut-off of 0.849 stratified participants high- and low-diversity groups; children above this threshold exhibited greater improvements in MetScore (*p* = 0.028), SBP (*p* = 0.043), and in HDL-cholesterol (*p* = 0.028).

**Conclusion:**

Higher baseline gut microbiota diversity and specific microbial signatures, particularly *Faecalibacterium* abundance, predicted better outcomes in childhood obesity interventions. These findings support the potential use of microbiota profiling to guide personalised treatment strategies. Further research is needed to optimise interventions.

**Trial registration:** clinicaltrials.gov NCT03749291.

## GRAPHICAL ABSTRACT

**Figure:**
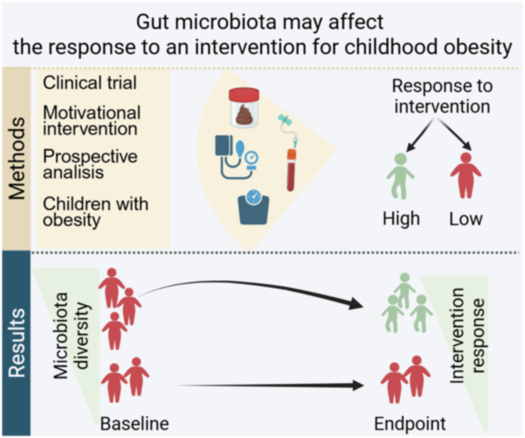

## Introduction

Dietary habits, together with physical activity, are among the most significant modifiable factors influencing the development of obesity.^1^^,^^2^ Consequently, the efforts to treat and prevent obesity have primarily focused on lifestyle programs that include dietary recommendations aimed at reducing body weight and improving metabolic health, in both adults and children.^3^ However, considerable inter-individual variability in treatment responses has been consistently observed.^4^ This variability underscores the importance of personalized nutrition, which seeks to tailor obesity interventions to the specific needs of individuals or groups by considering both host-specific and external factors that may influence the responsiveness to interventions.^5^

The gut microbiota is involved in numerous functions within the human body, including energy extraction, regulation of the host’s immunity and metabolism.^6^ For this reason, it has been proposed as an important player in the development of obesity and its associated comorbidities.^6^^,^^7^ Specific gut microbiota profiles, characterized by features such as low bacterial richness or reduced abundances of *Akkermansia*^8^^,^^9^, have been linked to predisposition to obesity-related cardiometabolic disorders.^10^

Several studies in both adults and children have examined how dietary modifications influence gut microbiota composition, thereby contributing to attenuation of obesity-related metabolic risk factors.^11^ Ghosh et al. demonstrated that adherence to the Mediterranean diet could increase the abundance of beneficial taxa such as *Faecalibacterium prausnitzii, Eubacterium and Roseburia,* which have been inversely associated with inflammatory markers.^12^ Similarly, in children, it was reported that a high-fiber diet intervention promoted the growth of specific strains of *Faecalibacterium, Bifidobacterium and Clostridium,* while reducing levels of *Bacteroides,* highlighting the strong influence of diet on microbiota composition.^13^

However, the gut microbiota is also known to play essential roles in nutrient absorption, energy storage and harvest from food intake. Recent studies have observed that individual responses to dietary interventions may be influenced by the pre-existing microbiota composition.^14^^,^^15^ In pediatric populations, a recent work by Zöggeler et al. has shown that specific gut microbiome profiles in children with obesity are associated with the progression of MASLD and MASH, with machine-learning models achieving high accuracy using microbial data.^16^

Advances in computational modeling have enabled the integration of biological data with microbial profiles to predict individual responses to specific interventions. For instance, a comprehensive study incorporating anthropometric variables, dietary habits, physical activity, blood parameters, and gut microbiota data from a cohort of 800 adults showed that incorporating gut microbiota profiles to anthropometric, lifestyle and biochemical parameters significantly improved the accuracy of machine-learning algorithms predicting postprandial glycaemic responses.^17^ Similarly, microbial richness and baseline gut microbiota composition have been shown to predict the effectiveness of weight-loss interventions in adults with obesity, with associations observed for both improvements in inflammatory profiles and reductions in waist circumference.^14^^,^^18^ Furthermore, the presence of specific taxa such as *Akkermansia muciniphila* has been linked to favorable metabolic outcomes after calorie restriction.^9^

These findings underscore the significant role of gut microbiota as a predictive biomarker of response to dietary and lifestyle interventions aimed at improving obesity and metabolic health. To date, limited research has examined whether gut microbiota composition can predict the effectiveness of nutritional interventions in children and adolescents.

Given the dynamic development of the gut microbiome during childhood and adolescence, understanding its role in modulating intervention outcomes may be essential for personalizing obesity management in these age groups. Therefore, the present study aimed to investigate whether gut microbiota composition could serve as a predictive marker of responsiveness to a structured weight-loss intervention in a cohort of children and adolescents with obesity.

## Materials and methods

The procedures for anthropometric and biochemical measurements, cardiometabolic risk score calculation and fecal sample collection and sequencing have been described in detail in our previous publication,^19^ and detailed further in the first author’s doctoral thesis.^20^

### Study design and participants

This longitudinal observational study was a secondary analysis of a randomized clinical trial that analyzed a weight-loss intervention for children with obesity. The present work includes baseline and final anthropometric and biochemical measurements, as well as baseline gut microbiota composition of participants who successfully completed the Obemat2.0 clinical trial^21‐23^ (clinicaltrials.gov NCT03749200) and participated in the Microbekids study (clinicaltrials.gov NCT03749291).

A total of 303 children with obesity (163 males; 140 females), aged between 8 and 14 years, were recruited from June 2016 to March 2018 from primary healthcare centers in Camp de Tarragona to take part in the Obemat2.0 clinical trial. Obesity was defined as BMI ≥ 97^th^ percentile according to criteria established by Hernandez et al.^24^ as recommended by the National Clinical Practice Guidelines.

Exclusion criteria were the presence of eating disorders, unavailability of families to attend at scheduled visits, concurrent participation in another clinical trial, presence of endocrine disorders (such as growth hormone deficiency, hypothyroidism, Cushing’s syndrome, precocious puberty or others) and lack of command in local languages.

The Obemat2.0 design was a randomized, non-blinded clustered intervention trial for treating children with obesity for 12 (+3) months. Briefly, the study included two arms (control and intervention). Children assigned to the control group received advice based on the “Guidelines for Clinical Practice on the Prevention and Treatment of Childhood and Adolescent Obesity” of the Spanish National Health System, which include explanations on balanced dietary patterns and physical activity recommendations.

The intervention arm consisted of patients who received a structured multicomponent intervention delivered over 11 visits. Each visit followed a similar format, comprising a specific topic for discussion and an at-home task. The session topics included information on obesity, guidance for food shopping, dietary balance and healthy menu planning, physical activity, strategies to manage anxiety, healthy lifestyle habits, breakfast and mid-afternoon snack recommendation, portion size, and a balanced distribution and balance of lunch and dinner. Moreover, they attended to a three group workshops including “Strategies to increase physical activity, Food products labeling and recommended food portions and cooking methods, a workshop performed at the kitchen.

Healthcare providers of the two groups received a short 4-hour training session on good clinical practice and the provision of diet and physical activity recommendations. In addition, healthcare professionals working with the intervention group received a 12-hour training program on structural motivational interviewing and were provided with educational materials, combined with group-based therapy tools, and eHealth resources.

Baseline and final assessments were conducted at Hospital Universitari Joan XXIII de Tarragona and Hospital Universitari Sant Joan de Reus, while the treatment itself was delivered in primary care centers across the area. Further details of the trial protocol, intervention, and main outcome results have been previously published.^22^^,^^23^

During the baseline and final assessments, the participants were invited to take part in a voluntary fecal and blood sample extraction as part of the registered collection of biological samples (COLOBEPED, reference C.0004585).

Participants who followed the treatment *Per Protocol* (attending to at least nine of the eleven visits, as well as the baseline and the final assessments), participated in the baseline fecal sample collection and in both, baseline and final blood extraction with no missing anthropometric (body mass index) data, were included in the present analyzes.

### Anthropometry, blood pressure and biochemical parameters

Body weight was measured using a digital scale (SECA 769, precision 50 g) while participants wore underwear. Standing height was measured with a wall-mounted stadiometer (SECA 216, precision 0.1 cm), and waist circumference was measured at the end of expiration, midway between the iliac crest and lower rib with a Holtain non-extensible tape (precision 0.1 cm). Body mass index (BMI) was calculated as weight divided by height squared (kg/m^2^), and BMI z-score was calculated according to the World Health Organization references.^25^

Fasting blood samples were collected by trained nurses. High-density lipoprotein cholesterol (HDL-cholesterol) (mg/dL), triglycerides (mg/dL), glucose (mg/dL) and insulin (µIU/mL) were determined at the certified laboratories of local study sites using routine clinical diagnostic methods. Insulin concentration was quantified by immunoradiometric assays, and the remaining parameters were measured using enzymatic methods. The Homeostasis Model Assessment of Insulin Resistance (HOMA-IR) was calculated as follows: HOMA-IR = (Insulin (µIU/mL)) x Glucose (mmol/L))/22.5.^26^

At least 20 minutes after arriving at the study center, study personnel measured systolic blood pressure (SBP) and diastolic blood pressure (DBP) (mmHg). Blood pressure was assessed in duplicate (with a time slot of 5 min between measures) using a Dinamap Pro 100 device on the left arm, while the child was sitting with the arm resting comfortably. The mean value of the two readings was used for the analysis.

Biochemical parameters, SBP, and DBP were standardized as z-scores according to the reference data from Stavnsbo et al.^27^

### Cardiometabolic health

The cardiometabolic risk score (MetScore) was calculated as a continuous variable based on the methodology reported by Eisenmann et al.^28^ It was computed by summing the standardized SBP, DBP, triglycerides, HOMA-IR, waist and HDL-cholesterol z-scores calculated according to Stavnsbo et al. references.^27^ The HDL-cholesterol z-score was multiplied by −1 to account for its inverse relationship with cardiometabolic risk. Higher MetScore values indicated a less favorable cardiometabolic profile.

### Fecal DNA collection, extraction and sequencing

All participants who agreed to provide fecal samples received a kit consisting of a stool sampler, a temperature isolation bag, and an ice box. Samples were collected one day before the scheduled visit, were stored at home at −20 °C, and were subsequently transported frozen to the study center. On the day of the visit, the samples were immediately placed in −80 °C freezers at the respective Biobanks.

DNA was extracted from approximately 200 mg of stool samples using the MagAttract PowerSoil DNA kit (Qiagen, Venlo, Netherlands) following the manufacturer’s protocol. Extractions were performed using the KingFisher Duo Primer Purification System (Thermo Fischer Scientific Inc, Waltham, MA, USA). Extractions were carried out at the ICTS infrastructure using equipment from the Center for Omic Sciences (COS), Joint Unit of the Universitat Rovira i Virgili and Eurecat.

Sequencing was performed at the FISABIO sequencing service (Valencia, Spain). DNA libraries were prepared according to the Illumina 16S rRNA Metagenomic Sequencing Library Preparation protocol (Code 15044233 Rev. A), targeting the variable V3 and V4 regions. The primers were selected according to Klindworth et al.^29^ Microbial genomic DNA (5 ng/μL in 10 mM Tris, pH 8.5) was used to initiate the protocol. The multiplexing step was performed with a Nextera XT Index Kit (FC-131-1096) (Illumina, San Diego, CA, USA). One μl of the PCR product was run on a Bioanalyzer DNA 1000 chip to verify the size (the expected size on a Bioanalyzer trace was ~550 bp). The libraries were sequenced with a 2 x 300bp paired-end run (MiSeq Reagent kit v3 (MS-102-3001)) on an Illumina MiSeq Sequencer, according to the manufacturer’s instructions. The quality assessment was performed using the PRINSEQ-lite program^30^ and sequences were selected with a minimum length of 50 bases. Sequence data were analyzed with QIIME2 pipeline by Bolyen et al.^31^ The metataxonomic analyzes were performed using the same pipeline, including the taxa plugin for taxonomic assignment. Denoising, paired-ends joining, and chimaera removal were performed using the DADA2 pipeline.^32^ Analyzes were conducted using paired-end sequence data. Taxonomic assignment was conducted using the Silva v138 database, including species-level classification obtained from the same database.^33^

### Response to the intervention

We assessed two response outcomes: BMI and MetScore improvements. Participants were categorized into two groups based on longitudinal changes in BMI z-score and MetScore during the study follow-up. Groups were defined as higher response (BMI-HR or MetScore-HR) and lower response (BMI-LR or MetScore-LR).

Children were classified as BMI-HR or MetScore-HR groups if they achieved a reduction greater than the median change. The median reduction was −0.37 [−0.57 to 0.10] for BMI z-score and −1.21 [−2.72 to 0.86] for MetScore. Participants with smaller improvements were classified as BMI-LR and MetScore-LR. More negative values indicated greater improvement in metabolic health and BMI z-score.

### Statistical analysis

The Kolmogorov-Smirnov test was used to assess the normality of all variables. Continuous variables are presented as median and interquartile range (25^th^-75^th^ percentiles) if non-normally distributed. Normally distributed variables are presented as mean and standard deviation. Categorical variables are reported as frequency and percentage (*n*, %).

The Kruskal-Wallis test was used to assess differences between higher-and lower-response groups. Outcomes included anthropometric measurements, biochemical parameters, relative abundance of bacterial taxa and alpha diversity indices. The Wilcoxon test was applied to analyze paired differences between baseline and final assessments.

The processed ASV table, taxonomic assignments and sample metadata were imported into RStudio and combined into a *phyloseq* object for integrated data management and analysis.^34^ ASVs not present in any sample were first removed, followed by the application of a prevalence filter using the *metagMisc* package,^35^ excluding ASVs present in fewer than 5% of the samples. For each sample, relative abundance (%) was calculated. Alpha diversity indices were calculated as Shannon, Simpson (expressed as inverse Simpson index), Observed species, ACE and Fisher. Linear regression models were used to assess associations between alpha-diversity indices and response to the intervention, adjusting for baseline age, sex, intervention group, and BMI z-score change.

To identify differentially abundant bacterial taxa between higher and lower response, we applied Linear Discriminant Analysis (LDA) effect size (LEfSe) at the ASV level. LEfSe was run using a *p*-value threshold of 0.05 and an LDA score cut-off of 2.0. Results were visualized using bar plots highlighting bacterial taxa enriched in each response group.

Analysis of Composition of Microbiome with BIAS Correction (ANCOM-BC2) was performed at ASV level to estimate bias-corrected log fold changes. Taxa with a prevalence below 10% were excluded. *P*-values were adjusted using Benjamini-Hochberg procedure. Models were adjusted for baseline age and BMI z-score, sex and intervention group. The reference category was the “higher response” group, and default ANCOM-BC2 parameters were applied.

To explore the predictive potential of microbiota and clinical features, we trained a Random Forest classification model using the *caret* package in RStudio. Microbiota features were aggregated at genus level prior to analysis. Feature selection was first performed using a Random Forest model including all genera, and the top 10% of gut microbiota features ranked by Mean Decrease in Accuracy (MDA)^36^ were retained and used to train the final classification model.

The model was trained to classify participants into high and low responders to the intervention based on selected bacteria, the Simpson alpha diversity index, and clinical covariates (baseline age, sex, and baseline BMI for the models trying to predict the BMI improvement, and baseline MetScore for the models trying to predict MetScore improvement). A 3-fold cross-validation (3 folds, 3 repeats) was used. Model performance was evaluated using the area under the receiver operating characteristic curve (ROC AUC). Predictor importance was assessed using mean decrease in the Gini index. The model performance was further evaluated using the statistical parameters derived from the confusion matrices. Finally, LEfSE and Random Forest results were compared to identify overlapping genera with both statistical and predictive relevance.

Spearman’s rank correlations were used to identify simple associations between different alpha diversity indices (Shannon and Simpson index), baseline bacterial abundances, and metabolic health parameters.

To evaluate the potential applicability of gut microbiota diversity as a standalone predictive biomarker, receiver operating characteristic (ROC) analysis was performed to determine an optimal cut-off value for the Simpson diversity index, which showed the strongest association with MetScore response. This threshold was then used to classify children into high- or low-diversity groups prior to the intervention. Health outcome improvements were then compared between groups using Kruskal-Wallis tests. Binary logistic regression models were conducted to quantify the odds of a poor response to the intervention based on diversity status. Models were adjusted for sex, baseline age, intervention group and BMI z-score change.

All data management and statistical analyzes were conducted using RStudio 2025.05.0.^37^

### Ethics

The study followed the rules of the Declaration of Helsinki^38^ and was approved by the ethics committees responsible for the activity of all the involved study centers (CEIM Institut d’Investigació Sanitària Pere Virgili). All parents or legal guardians signed the informed consent before study enrollment, as well as for participating in the registered samples collection (COLOBEPED, reference C.0004585), and children aged 12 years or above signed an informed assent form to participate.

## Results

### Characteristics of the study participants

Seventy-two children with obesity provided a baseline fecal sample and completed blood extraction at both the baseline and final assessments. Of these, forty-one participants (57% boys and 43% girls) from the Microbekids study attended at least nine of the eleven Obemat2.0 intervention visits and were included in the final analysis (**Supplementary Figure 1**).

Table 1 presents the baseline and final characteristics of all study participants according to their response to the intervention. The median age of all the participants was 10.0 [IQR: 9.0 to 12.0] years, and the median BMI z-score was 2.60 [IQR: 2.14 to 2.78] at the study entry. No significant differences were observed at baseline between higher and lower-response groups.

**Table 1.** Characteristics of the study cohort at baseline and final study visits classified by the degree of response to the intervention.

		BMI response			MetScore response		
	ALL	Higher Response	Lower Response	*P*-value	Higher Response	Lower Response	*P*-value
N	41	21	20		21	20	0.039
Sex (boy/girl)	23/18	12/9	11/9		8/13	15/5	
**Baseline**							
Age (y)	10.0 [9.00;12.0]	10.0 [9.00;11.0]	11.0 [9.00;12.0]	0.288	10.0 [10.0;12.0]	10.0 [8.75;12.0]	0.482
BMI z-score	2.60 [2.14;2.78]	2.32 [2.08;2.91]	2.62 [2.48;2.72]	0.235	2.62 [2.29;2.91]	2.57 [2.12;2.73]	0.611
Waist z-score	1.86 [1.46;2.39]	1.86 [1.35;2.39]	1.91 [1.57;2.17]	0.969	2.03 [1.65;2.39]	1.69 [1.26;2.15]	0.192
Triglycerides z	0.29 [−0.31;0.68]	−0.22 [−0.31;0.40]	0.45 [−0.06;0.76]	0.188	0.29 [−0.29;0.47]	0.17 [−0.48;0.76]	0.979
HDL-c z-score	−0.40 [−0.93;0.21]	−0.15 [−1.01;0.38]	−0.50 [−0.88; −0.04]	0.489	−0.58 [−0.93;0.09]	−0.23 [−0.84;0.28]	0.397
HOMA-IR z-score	0.60 [0.37;1.31]	0.48 [0.10;0.98]	0.92 [0.41;1.45]	0.130	0.71 [0.48;1.43]	0.46 [−0.09;1.21]	0.060
SBP z-score	0.48 [−0.44;1.28]	0.48 [−0.44;0.82]	0.54 [−0.24;1.29]	0.744	0.53 [0.15;1.28]	0.45 [−0.55;0.86]	0.375
DBP z-score	−0.11 [−0.51;0.37]	0.08 [−0.58;0.29]	−0.15 [−0.43;0.63]	0.657	−0.08 [−0.71;0.31]	−0.12 [−0.40;0.43]	0.629
MetScore	4.00 [1.97;5.75]	3.46 [1.22;5.48]	4.61 [3.33;5.77]	0.211	4.59 [1.98;6.11]	3.52 [1.87;4.75]	0.183
**Final assessment**							
Age (y)	11.0 [10.0;13.0]	11.0 [10.0;12.0]	12.0 [10.0;13.0]	0.265	11.0 [11.0;13.0]	11.0 [9.75;13.0]	0.533
BMI z-score	2.17 [1.70;2.67]	1.76 [1.45;2.09]	2.56 [2.19;2.71]	**0.001**	2.09 [1.70;2.69]	2.21 [1.74;2.65]	0.979
Waist z-score	1.50 [1.02;2.15]	0.20 [0.92;1.63]	1.70 [1.40;2.52]	**0.005**	1.50 [1.14;2.14]	1.53 [0.99;2.19]	0.764
Triglycerides	−0.04 [−0.52;0.44]	−0.22 [−0.54;0.04]	0.21 [−0.07;0.76]	**0.039**	−0.22 [−0.75;0.25]	0.13 [−0.12;1.64]	**0.017**
HDL-c z-score	−0.46 [−0.80;0.14]	−0.06 [−0.60;0.68]	−0.56 [−0.84; −0.16]	0.159	0.35 [−0.60;0.34]	−0.54 [−0.84; −0.01]	0.328
HOMA-IR z-score	0.16 [−0.38;0.63]	−0.20 [−0.62;0.34]	0.39 [0.09;0.70]	0.068	0.16 [−0.40;0.61]	0.17 [−0.31;0.67]	0.715
SBP z-score	0.13 [−0.28;0.92]	0.13 [−0.25;0.50]	0.44 [−0.40;1.56]	0.442	−0.10 [−1.01;0.42]	0.63 [0.03;1.35]	**0.006**
DBP z-score	0.40 [−0.10;0.84]	0.21 [−0.87;0.84]	0.41 [0.17;0.80]	0.328	0.04 [−0.87;0.66]	0.53 [0.39;1.11]	**0.012**
MetScore	3.14 [1.08;4.86]	1.70 [0.69;3.49]	4.30 [2.23;5.93]	**0.018**	1.70 [0.16;3.49]	4.30 [2.46;6.80]	**0.008**
MetScore difference^a^	−1.13 [−2.24;1.00]	−1.36 [−3.34;0.34]	0.29 [−1.37;1.10]	0.112	−2.24 [−3.62; −1.37]	1.02 [0.33;2.64]	**<0.001**
BMI z-score difference^b^	−0.37 [−0.57; −0.10]	−0.57 [−0.80; −0.53]	−0.09 [−0.25;0.00]	**<0.001**	−0.40 [−0.55; −0.26]	−0.27 [−0.61; −0.04]	0.361

BMI: Body mass index; HDL-c: High-density lipoprotein cholesterol; MetScore: Metabolic risk score. *P*-value calculated by paired samples Wilcoxon test.

a MetScore reduction Final MetScore—Baseline MetScore.

b BMI z-score difference = Final BMI z-score—Baseline BMI z-score. A greater negative value indicates a greater reduction in both, BMI and MetScore.

At the end of the intervention, children classified as BMI-LR showed significantly higher BMI z-score, waist circumference z-score, triglyceride concentrations z-score and MetScore. Similarly, those in the MetScore-LR group presented significantly higher triglycerides, systolic and diastolic blood pressure and higher MetScore. Significant changes were observed following the treatment, including a significant change in BMI z-score by a reduction of more than 0.4 SD (*p* < 0.001), waist circumference by −0.33 SD (*p* = 0.067) and HOMA-IR by −0.42 SD (*p* = 0.002). A slight increase in DBP was observed.

The median differences between the final and baseline MetScore and BMI z-score values were −1.21 [−2.72 to 0.86] and −0.37 [−0.57 to −0.10], respectively.

### Gut microbiota characteristics according to the response to the intervention

Alpha diversity indices according to MetScore and BMI response groups (HR vs LR) are summarized in **Supplementary Table 1**. We observed a trend towards higher alpha-diversity indices in the MetScore-HR group, as reflected by ACE and Shannon indices, and a statistically significant difference in the Simpson index. In the BMI-HR group, diversity indices were generally higher than in BMI-LR, although none of the differences reached statistical significance.

ANCOM-BC2 identified two taxa significantly associated with the BMI (FDR < 0.05): an ASV assigned to the *Eubacterium coprostanoligenes group_(s_gut metagenome)* and the *Ruminococcus* genus. Overall 84 ASVs were significantly associated with MetScore response (FDR *p* < 0.05). Several of these ASVs belonged to the genera *Eubacterium coprostanoligenes group, Bacteroides, Bifidobacterium or Ruminococcus* (**Supplementary Table 3).**

**Figure 1.**
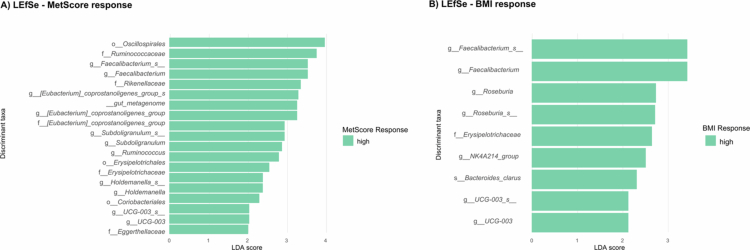
Linear discriminant analysis (LDA) integrated with effect size (LEfSe). A&B) These bar plots illustrate LDA scores, representing the difference in relative bacterial abundances between individuals who had a high response (HR) from those who had a low response (LR) to the intervention in both outcomes, MetScore (A) and BMI (B). Only bacteria with LDA scores greater than 2 and significant Wilcoxon test results are shown, indicating a significant difference between groups.

LEfSE analysis was conducted to identify the most differentially abundant taxa between higher and lower responders to both MetScore and BMI (Figure 1A & 1B). We identified 20 and 11 bacterial taxa associated with the MetScore-HR and BMI-HR groups, respectively. No bacterial taxa exhibited an LDA score greater than 2 in the lower-response groups.

Figure 1A shows taxa enriched in the MetScore-HR group (LDA > 2) compared to the MetScore-LR group. These included the orders Oscillospirales, Erysipelotrichales and Coriobacteriales, the families Ruminococcaceae, Rikenellaceae, and Erysipelotrichaceae, the genus *Faecalibacterium, Eubacterium coprostanoligenes group*, *Subdoligranulum*, *Ruminococcus*, *Holdemanella and UCG−003*, an unclassified species of the same genus; and the specific *Eubacterium coprostanoligenes group gut metagenome*. Figure 1B displays taxa enriched in BMI-HR (LDA > 2), with highlighted genera including *Faecalibacterium* and *Roseburia*. Relative abundance distributions for taxa identified by LEfSe are shown in **Supplementary Figure 2A and 2B.**

Different genera identified by LEfSE in the MetScore-HR group showed concordant directional log fold-change estimates in ANCOM-BC2, including ASVs belonging to the genera *Ruminococcus* and the E*ubacterium coprostanoligenes group*.

#### Baseline gut microbiota profile as a predictor of the response to the intervention

**Figure 2.**
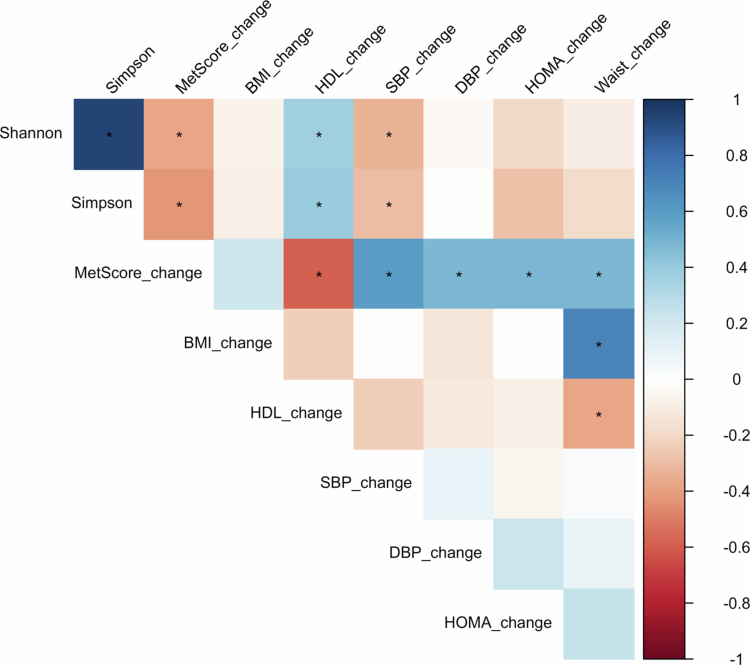
Correlation matrix between metabolic parameters and diversity indices. Each box displays the Spearman correlation coefficient between the variables. Larger dots indicate a higher correlation coefficient, with red dots representing negative associations and blue dots representing positive associations. Statistical significance is represented as (*). Variables change: change between baseline and final visits. MetScore: Metabolic risk score summary of z-scores of cardiovascular risk factors.; BMIz: body mass index z-score; HDL: high density lipoproteins; SBP: systolic blood pressure; DBP: diastolic blood pressure; HOMA: homeostasis model of insulin resistance index; waist: waist circumference.

Statistically significant associations were observed between baseline Simpson and Shannon indices and post-intervention improvements in MetScore (Rho = −0.43, *p* = 0.005 and Rho = −0.38, *p* = 0.01, respectively), SBP (Rho = −0.38, *p* = 0.01 and Rho = −0.39, *p* = 0.01, respectively), and HDL-cholesterol (Rho = 0.53, *p* < 0.001 and Rho = 0.46, *p* = 0.002, respectively) (Figure 2). No associations were observed between baseline gut microbiota diversity indices and the BMI z-score response.

Linear regression models showed that the Shannon and Simpson indices, adjusted for clinical covariates, explained approximately 30% of the variance in MetScore response (Table 2). Associations with HDL-cholesterol improvement remained statistically significant after adjustment.

**Table 2.** Results of the linear regression models for gut microbiota diversity (Shannon and Simpson indices) predicting the response (metabolic health parameters) to the intervention.

			Predicted variables			
	MetScore change		HDL-c change		SBP change	
Predictors	*B (95% CI)*	*p*	*B (95% CI)*	*p*	*B (95% CI)*	*p*
**Shannon index**	**−2.95(−5.00** **–0.90)**	**0.006**	**0.66(0.23** **–1.09)**	**0.004**	**−0.89(−1.93** **–0.16)**	**0.094**
Intervention group [Intervention]	−0.64(−2.30–1.02)	0.438	0.16(−0.18–0.51)	0.347	−0.01(−0.85–0.84)	0.988
Sex [girl]	−1.28(−2.89–0.33)	0.116	0.04(−0.30–0.38)	0.826	−0.13(−0.96–0.69)	0.742
Baseline age	−0.01(−0.06–0.03)	0.535	0.00(−0.01–0.01)	0.383	0.02(−0.00–0.04)	0.102
BMIz difference	1.29(−1.35–3.94)	0.327	−0.34(−0.89–0.22)	0.225	−0.43(−1.78–0.92)	0.518
Observations	41		41		41	
R^2^/R^2^ adjusted	0.285/0.183		0.263/0.158		0.176/0.058	
**Simpson index**	**−13.70(−22.74** **–4.65)**	**0.004**	**2.89(0.96** **–4.82)**	**0.004**	**−3.34(−8.06** **–1.38)**	**0.160**
Intervention group [Intervention]	−0.76(−2.42–0.90)	0.359	0.16(−0.18–0.51)	0.347	−0.00(−0.87–0.86)	0.996
Sex [girl]	−1.02(−2.63–0.59)	0.205	0.04(−0.30–0.38)	0.826	−0.08(−0.92–0.76)	0.857
Baseline age	−0.01(−0.06–0.03)	0.524	0.00(−0.01–0.01)	0.383	0.02(−0.00–0.04)	0.097
BMIz difference	1.21(−1.41–3.83)	0.354	−0.34(−0.89–0.22)	0.225	−0.43(−1.80–0.93)	0.525
Observations *(n)*	41		41		41	
R^2^/R^2^ adjusted	0.300/0.200		0.163/0.157		0.156/0.035	

BMI: Body mass index; HDL-c: High-density lipoprotein cholesterol; MetScore: Metabolic risk score; SBP: Systolic blood pressure. BMI z-score difference = Final BMI z-score – Baseline BMI z-score. A greater negative value indicates a greater reduction in both BMI and MetScore.

Although several bacterial taxa were associated with MetScore and BMI improvement (**Supplementary Table 2)**, only the order Oscillospirales remained significantly associated with MetScore improvement after false-discovery-rate correction.

A Random Forest classifier was trained as a supervised learning model. Feature selection was performed using variable importance scores, retaining the top 10% of bacterial genera and reducing the feature set from 177 to 19 genera. These genera, together with the Simpson diversity index and clinical covariates (age, sex and baseline BMI or MetScore), were included in Random Forest models, resulting in improved model performance and accuracy.

**Figure 3.**
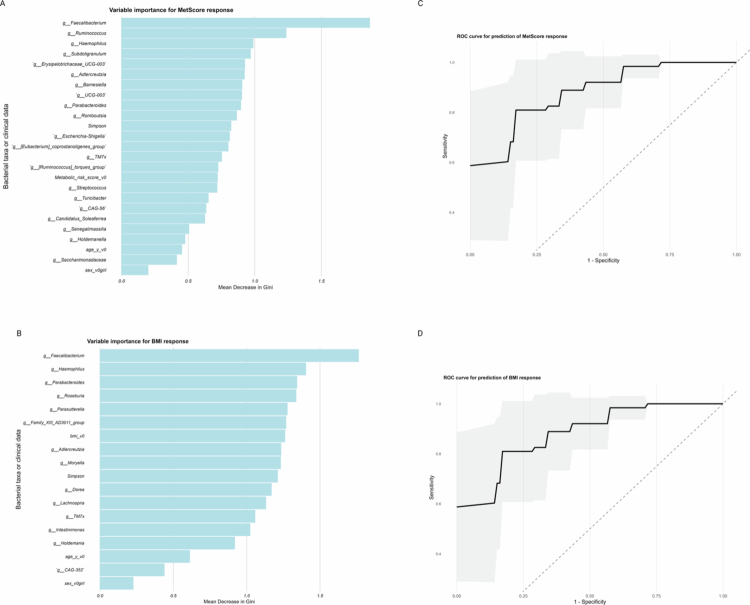
Predictive importance of gut microbiota and clinical variables in intervention response. A&B panels show the top-ranked variables identified by the Random Forest model for predicting (A) MetScore improvement and (B) BMI z-score reduction after a structured lifestyle intervention. The x-axis represents the mean decrease in the Gini index, indicating the variable importance. C&D show the receiver operating characteristics (ROC) curves for the Random Forest models predicting (c) MetScore improvement and (D) BMI z-score improvement. The shaded areas represent 95% confidence intervals.

The MetScore model achieved an ROC AUC of 0.876 (95% CI: 0.812-0.940), with 72% and sensitivity 86% specificity. Similarly, the BMI model achieved an ROC AUC of 0.873 (95% CI 0.807-0.929), with 73% sensitivity and 87% specificity. McNemar’s test was not statistically significant for either model, indicating balanced distribution of classification errors (Figure 3C and Figure 3D).

Variable importance analysis, based on the mean decrease in Gini index identified *Faecalibacterium, Ruminococcus, Haemophilus and Subdoligranulum* as the strongest predictors of MetScore improvement, with Simpson’s index ranking mid-range among the most influential variables (Figure 3A). In the BMI model, *Faecalibacterium, Haemophilus, Parabacteroides and Roseburia* were the top contributors, with both baseline BMI and Simpson’s index ranking within the top 10 predictors (Figure 3B).

**Figure 4.**
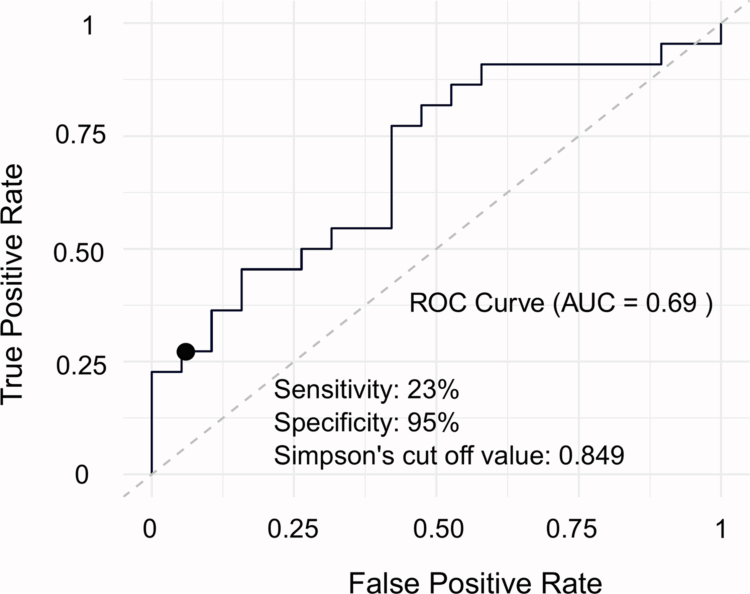
ROC curve for Simpson cut-off value. This ROC curve illustrates an optimal baseline Simpson cut-off value to detect those participants who will have a MetScore-LR (low response to the intervention).

Given the consistent predictive contribution of Simpson diversity in both models, a ROC analysis was performed to determine a baseline Simpson threshold capable of distinguishing lower MetScore responders. The analysis revealed an AUC of 0.690, and the optimal cut-off value identified was 0.849, with 23% sensitivity and 94% specificity (Figure 4). Seven children were classified below this threshold and showed significantly lower improvement in MetScore (*p* = 0.014) compared to those with a higher Simpson value (Table 3). The same pattern was observed for SBP and HDL-cholesterol, but not association was found with BMI z-score.

**Table 3.** The difference in the cardiometabolic parameters in children with high and low gut microbiota diversity according to the proposed threshold.

Outcome variables (z-score change over time)	Low diversity (Simpson ≤ 0.849) *n* = 7	*High diversity* (Simpson > 0.849) *n* = 34	*p-value*
Body mass index	−0.53 [−0.58; −0.16]	−0.36 [−0.55; −0.12]	0.835
Triglycerides	0.09 [0.00;0.74]	−0.16 [−0.56;0.19]	0.111
High-density lipoprotein cholesterol	−0.41 [−0.64;0.07]	0.23 [−0.20;0.50]	**0.043**
Systolic blood pressure	0.45 [0.06;0.86]	−0.52 [−1.16;0.27]	**0.028**
Diastolic blood pressure	0.24 [0.07;0.60]	0.35 [−0.31;0.92]	0.959
HOMA-IR	−0.19 [−0.66;0.09]	−0.65 [−1.23; −0.16]	0.268
Waist circumference	−0.15 [−0.33;0.13]	−0.29 [−0.63;0.12]	0.467
MetScore	0.97 [0.11;2.84]	−1.31 [−3.58;0.52]	**0.014**

Variable change is the difference between the final and baseline parameters. Index; HOMA-IR: Homeostasis Model Assessment for Insulin Resistance; MetScore: Metabolic risk score; Negative values represent a reduction of the specific parameter.

**Figure 5.**
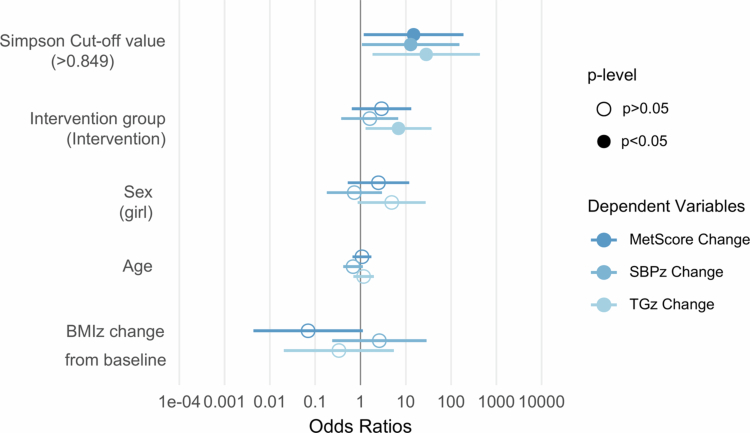
Forest plot of logistic regression models on cardiometabolic outcomes improvement according to microbiota diversity level. The horizontal bar represents 95% of the confidence interval. Filled circles represented statistically significant results, while open circles represented non-significant results.

Figure 5 shows the binary logistic regression models assessing response to the treatment according to the diversity threshold. After adjusting for age, sex, BMI z-score change and intervention group, participants classified as a low diversity group (Simpson index < 0.849) had approximately nine-fold higher odds of showing a poor response to the treatment compared with those in the higher diversity group (OR: 9.21, 95% CI: 1.63–75.95, *p* = 0.020). Similar trends were observed for reduction in triglycerides (OR: 0.04, 95% CI: 0.00–0.36, *p* = 0.016) and SBP (OR: 0.08, 95% CI: 0.00–0.064, *p* = 0.043), indicating a consistent pattern. No association was observed with improvements in BMI.

## Discussion

Our findings show that baseline gut microbiota composition in children with obesity may serve as a useful predictor of treatment effectiveness. Using ANCOM-BC2, we identified several bacterial taxa that differed significantly between response groups at baseline, particularly with respect to MetScore improvement. Exploratory analyzes using LEfSe and Random Forest revealed consistent patterns. Both models suggested that greater reductions in MetScore were associated with higher baseline abundances of the *Oscillospirales* order, the Rikenellaceae and the Ruminococcaceae families, the *Eubacterium coprostanoligenes group,* and the genus *Faecalibacterium*. Additionally, greater improvements in BMI z-score were associated with higher abundances of *Faecalibacterium and Roseburia genera. Higher alpha diversity was also associated* with significantly greater improvements in cardiovascular health-related items.

Previous studies have identified specific bacterial taxa that may modulate the response to weight-loss interventions in adults.^9^^,^^17^^,^^39^ However, the particular role of different gut microbiota compositions in shaping treatment outcomes remains poorly understood. Dao et al.,^9^ for instance, reported that a higher baseline abundance *of Akkermansia muciniphila* was associated with greater improvement in insulin sensitivity and body fat distribution after a six-week restricted diet intervention. In our sample, a higher abundance of Rikenellaceae and Ruminococcaceae families was associated with greater improvement in the cardiometabolic risk score. These results may reflect beneficial microbial functions, such as increased fermentation and short-chain fatty acid production, which are known to have a positive influence on the host metabolism.

To further evaluate the predictive capacity of the baseline gut microbiota, we applied supervised machine-learning models (Random Forest models). Notably, the genus *Faecalibacterium* emerged as one of the strongest predictors of both metabolic and BMI response. This genus is a well-known butyrate producer with established anti-inflammatory properties.^40^^,^^41^ Its most abundant species, *Faecalibacterium prausnitzii,* has been described as a key regulator of gut health through its contributions to the intestinal barrier maintenance and immune modulation,^41^ and is recognized as one of the most important butyrate-producing bacteria in the human colon. Butyrate, the most abundant short-chain fatty acid (SCFA) derived from the fermentation of non-digestible carbohydrates in the colon, serves as a primary energy source for colonocytes, supports gut barrier integrity, and activates anti-inflammatory signaling pathways.^42^^,^^43^ A recent pediatric study found that combining Mediterranean diet recommendations with butyrate supplementation led to greater reductions in BMI compared to a placebo.^44^ Notably, baseline abundance of *F. prausnitzii* was associated with improved insulin sensitivity (as measured by HOMA-IR), in line with our observations and supporting the central role of this genus in metabolic health.

Previous studies have suggested that early-life gut microbiota composition may predict growth trajectories and the risk of overweight,^45^ and increasing evidence shows that gut microbiota can influence the response to dietary interventions in conditions such as inflammatory bowel syndrome.^46^ Chumpitazi et al.^46^ reported that children with inflammatory bowel syndrome who responded positively to a low FODMAP (fermentable oligosaccharides, disaccharides, monosaccharides, and polyols) diet had greater gut microbial richness and higher abundance of specific bacteria associated with carbohydrate fermentation and butyrate production.^47^ To our knowledge, few studies have explored the relationship between gut microbiota and weight loss in children.^48^ Nadal et al.^48^ observed that children with obesity who achieved a greater weight loss after treatment had higher baseline abundances of *Lactobacillus*.

We also observed that the *Eubacterium coprostanoligenes* group was associated with improvements in metabolic health. Members of this group can convert cholesterol into coprostanol,^49^ which is a poorly absorbed metabolite. Consistent with this, several studies have reported inverse associations between coprostanol levels and plasma cholesterol, suggesting that microbial cholesterol-to-coprostanol conversion may represent a potential strategy for cholesterol management in humans.^50^

Gut microbiota diversity, particularly measured by Simpson’s diversity index, emerged as a key variable predicting treatment success. We identified a potential cut-off value for the Simpson diversity index that, if replicated in future studies, could help to identify children at higher risk of poor response to intervention. Early identification of such children may enable clinicians to design tailored interventions, such as increasing gut microbiota diversity through prebiotics or butyrate supplementation before initiating lifestyle changes, thereby maximize the effectiveness of family-based efforts.

As previously mentioned, while the gut microbiome can influence intervention outcomes, but the intervention itself can also modify the gut microbiota. For instance, children on calorie-restricted diets have been shown to increase alpha diversity and enrich beneficial strains such as *Faecalibacterium.*^51^^,^^52^ Therefore, although we identified specific taxa associated with better response to the intervention, the variability of individual taxa and the complex ecological interactions within microbial communities may limit the ability to predict individual outcomes with high precision.

One potential criticism is that it could be argued that improvements in metabolic health are more likely attributed to the intervention recommendations rather than from baseline gut microbiota composition. The fact that our analysis was conducted on a subset of children who adhered to the treatment protocol, and that the results remained significant after adjusting for BMI loss, suggests that the observed effects are not only explained by the weight reduction, supporting a potential independent role of the gut microbiota in metabolic improvement.

One of the limitations of our work was the relatively small sample size of each group which could limit the generalization of our findings. However, it is worth highlighting that our results are consistent with previous findings in the literature, and this is the largest sample size of children whose gut microbiota has been analyzed and associated with health outcomes longitudinally, following an intervention. Efforts to address potential sources of bias were controlled for confounders.

Validating the predictive role of microbiota diversity and composition in independent cohorts accounting for different ages and countries is essential before it can be applied in clinical or public health settings. A potential further application of these findings is the development of personalized nutrition interventions, aimed at modulating the gut microbiota to enhance the treatment success. Although assessing microbiota diversity in children with obesity may be costly in clinical practice, it may be useful to identify individuals who are more likely to benefit from dietary interventions. This targeted approach could help optimize resources and reduce discouragement experienced by children and their families when interventions fail to produce results.

## Conclusions

In conclusion, our study highlights the potential of gut microbiota diversity, as well as specific bacteria such as *Faecalibacterium*, as predictive markers of metabolic health and BMI improvement in children following a structured lifestyle intervention. We propose a threshold for microbiota diversity that may help identify individuals who are less likely to respond to such interventions. However, external validation of this threshold in independent cohorts is required. Further research should aim to holistically integrate lifestyle factors, physiological parameters, and microbiota signatures to improve personalized treatment strategies for children with obesity.

## Supplementary Material

AlcazarM_GutMicrobiota_SupplementaryTables_1and2_clean.docx AlcazarM_GutMicrobiota_SupplementaryTables_1and2_clean.docx

Supplementary_Tables_3and4.xlsx Supplementary_Tables_3and4.xlsx

AlcazarM_GutMicrobiota_FigureS2.png Supplemental Material

AlcazarM_GutMicrobiota_FigureS1.png Supplemental Material

Supplementary_figures.docx Supplemental Material

## Data Availability

The data supporting the findings of this study are publicly available in the CORA Research Data Repository (https://doi.org/10.34810/data2706).

## Clinical trial registration

Motivation Approach for Childhood Obesity Treatment (OBEMAT): NCT02889406.

Motivational Intervention on the Gut Microbiota of Obese Children (MICROBEkids): NCT03749291.
