# Supplementary material for: Children’s gut microbiota predicts the efficacy of obesity treatment
Source: Gut Microbes. 2026 Feb 19;18(1):2631824. doi: 10.1080/19490976.2026.2631824 (PMC12928635; doi:10.1080/19490976.2026.2631824)
Supplement: AlcazarM_GutMicrobiota_SupplementaryTables_1and2_clean.docx [file KGMI_A_2631824_SM2788.docx]

**Supplementary Table 1. Alpha diversity index by group of response to the intervention.**

|  | Based on the MetScore Improvement | | | Based on the BMI z-score improvement | | |
| --- | --- | --- | --- | --- | --- | --- |
|  | MetScore-HR | MetScore-LR | P-value | High | Low | P-value |
| Observed species | 87.0 [81.0;96.5] | 85.5 [80.5;90.2] | 0.417 | 87.0 [82.0;97.0] | 84.5 [79.0;89.8] | 0.341 |
| ACE | 87.0 [81.0;96.5] | 85.5 [80.5;90.2] | 0.417 | 87.0 [82.0;97.0] | 84.5 [79.1;89.8] | 0.341 |
| Shannon | 3.05 [2.76;3.14] | 2.85 [2.56;3.01] | 0.056 | 3.04 [2.67;3.13] | 2.88 [2.70;2.97] | 0.419 |
| Simpson | **0.92 [0.89;0.93]** | **0.89 [0.85;0.91]** | **0.034** | 0.92 [0.86;0.93] | 0.90 [0.87;0.91] | 0.465 |
| InvSimpson | 12.2 [9.15;14.9] | 9.30 [6.61;11.2] | 0.034 | 12.2 [7.17;14.6] | 9.86 [7.42;11.0] | 0.465 |
| Fisher | 10.6 [8.98;11.1] | 10.2 [9.45;10.6] | 0.458 | 10.4 [10.0;11.1] | 10.1 [9.10;10.8] | 0.584 |
| BMI: Body mass index; MetScore: Metabolic risk score. P-values were calculated using the Kruskal-Wallis tests. HR represent those children with an improvement of MetScore or BMI higher than the median of the improvement from the baseline. LR represent those children with an improvement of the MetScore < than the median of the improvement from the baseline. | | | | | | |

**Supplementary Table 2.** Significant correlation between bacteria taxa and the response of the intervention.

|  | Rank | Bacteria | Corr | P value | P adjusted (FDR) |
| --- | --- | --- | --- | --- | --- |
| BMI z-score change | Phylum | Desulfobacterota | 0.40 | 0.009 | 0.12 |
|  | Class | Desulfovibrionia | 0.40 | 0.009 | 0.12 |
|  | Order | Desulfovibrionales | 0.40 | 0.009 | 0.12 |
|  | Family | Desulfovibrionaceae | 0.40 | 0.009 | 0.12 |
|  | Genus | Parasutterella | -0.32 | 0.039 | 0.44 |
|  |  | Bilophila | 0.39 | 0.01 | 0.30 |
|  |  | Adlercreutzia | -0.38 | 0.01 | 0.32 |
|  |  | Enterorhabdus | -0.38 | 0.01 | 0.31 |
|  |  | Pseudoflavonifractor | -0.34 | 0.02 | 0.40 |
|  | Species | Bacteroides vulgatus | 0.33 | 0.03 | 0.61 |
|  |  | Bifidobacterium bifidum | 0.33 | 0.03 | 0.60 |
|  |  | Bacteroides sp | 0.32 | 0.04 | 0.66 |
|  |  | Dialister propionicifaciens | -0.37 | 0.01 | 0.47 |
| MetScore change | Phylum | -- | -- | -- | -- |
|  | Class | Bacilli | -0.36 | 0.02 | 0.23 |
|  | Order | **Oscillospirales** | **-0.47** | **0.002** | **0.05** |
|  |  | Enterobacterales | 0.45 | 0.003 | 0.07 |
|  |  | Erysipelotrichales | -0.32 | 0.04 | 0.26 |
|  |  | Rhodospirillales | 0.34 | 0.03 | 0.23 |
|  | Family | Ruminococcaceae | -0.40 | 0.009 | 0.20 |
|  |  | Rikenellaceae | -0.34 | 0.03 | 0.31 |
|  |  | Bacteriodaceae | 0.37 | 0.02 | 0.26 |
|  |  | Eubacterium coprostanoligenes group | -0.51 | 0.0007 | 0.05 |
|  |  | Enterobacteriaceae | 0.44 | 0.003 | 0.12 |
|  |  | Eggerthellaceae | -0.40 | 0.009 | 0.20 |
|  |  | Saccharimonadales | -0.35 | 0.02 | 0.29 |
|  | Genus | *Faecalibacterium* | -0.41 | 0.007 | 0.25 |
|  |  | *Bacteroides* | 0.36 | 0.02 | 0.36 |
|  |  | *Eubacterium coprostanoligenes group* | -0.51 | 0.0006 | 0.07 |
|  |  | *Escherichia-Shigella* | 0.42 | 0.005 | 0.22 |
|  |  | *Ruminococcus* | -0.31 | 0.046 | 0.46 |
|  |  | *Terrisporobacter* | -0.35 | 0.03 | 0.38/ |
|  |  | *Rikenellaceae Rc9 gut group* | -0.37 | 0.01 | 0.32 |
|  |  | *Pseudoflavonifractor* | -0.36 | 0.01 | 0.36 |
|  |  | *Saccharimonadales* | -0.35 | 0.02 | 0.38 |
|  | Species | *Odoribacter splanchicus* | 0.42 | 0.006 | 0.32 |
|  |  | *Steptrococcus parasanguinis* | 0.41 | 0.006 | 0.33 |
|  |  | *Massiliomicrobiota timonesis* | -0.34 | 0.03 | 0.57 |
|  |  | *Candidatus Saccharibacteria* | -0.35 | 0.02 | 0.55 |
| *Change in BMI z-score is defined as the final BMI z-score minus the baseline BMI z-score. Change in MetScore is calculated as the final MetScore minus the baseline MetScore. | | | | | |
