# Supplementary figures and images for: Children’s gut microbiota predicts the efficacy of obesity treatment
Source: Gut Microbes. 2026 Feb 19;18(1):2631824. doi: 10.1080/19490976.2026.2631824 (PMC12928635; doi:10.1080/19490976.2026.2631824)

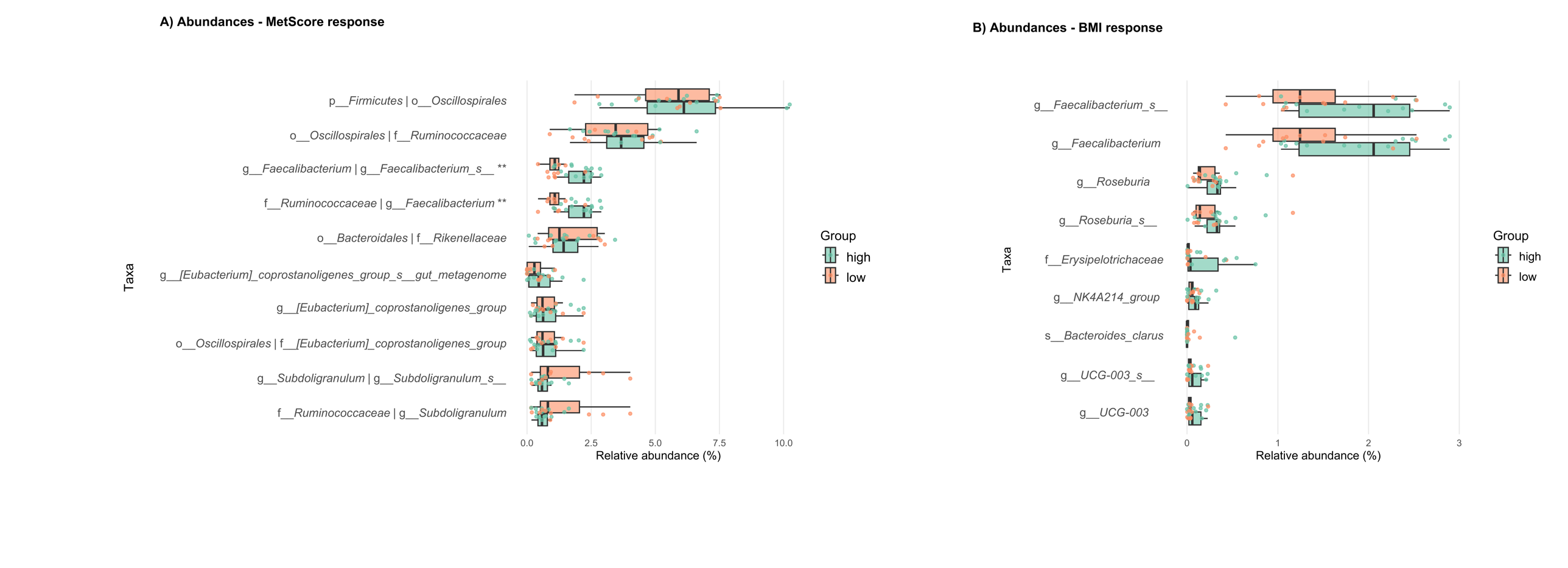

Supplement: AlcazarM_GutMicrobiota_FigureS2.png — Supplemental Material [file KGMI_A_2631824_SM3084.png]

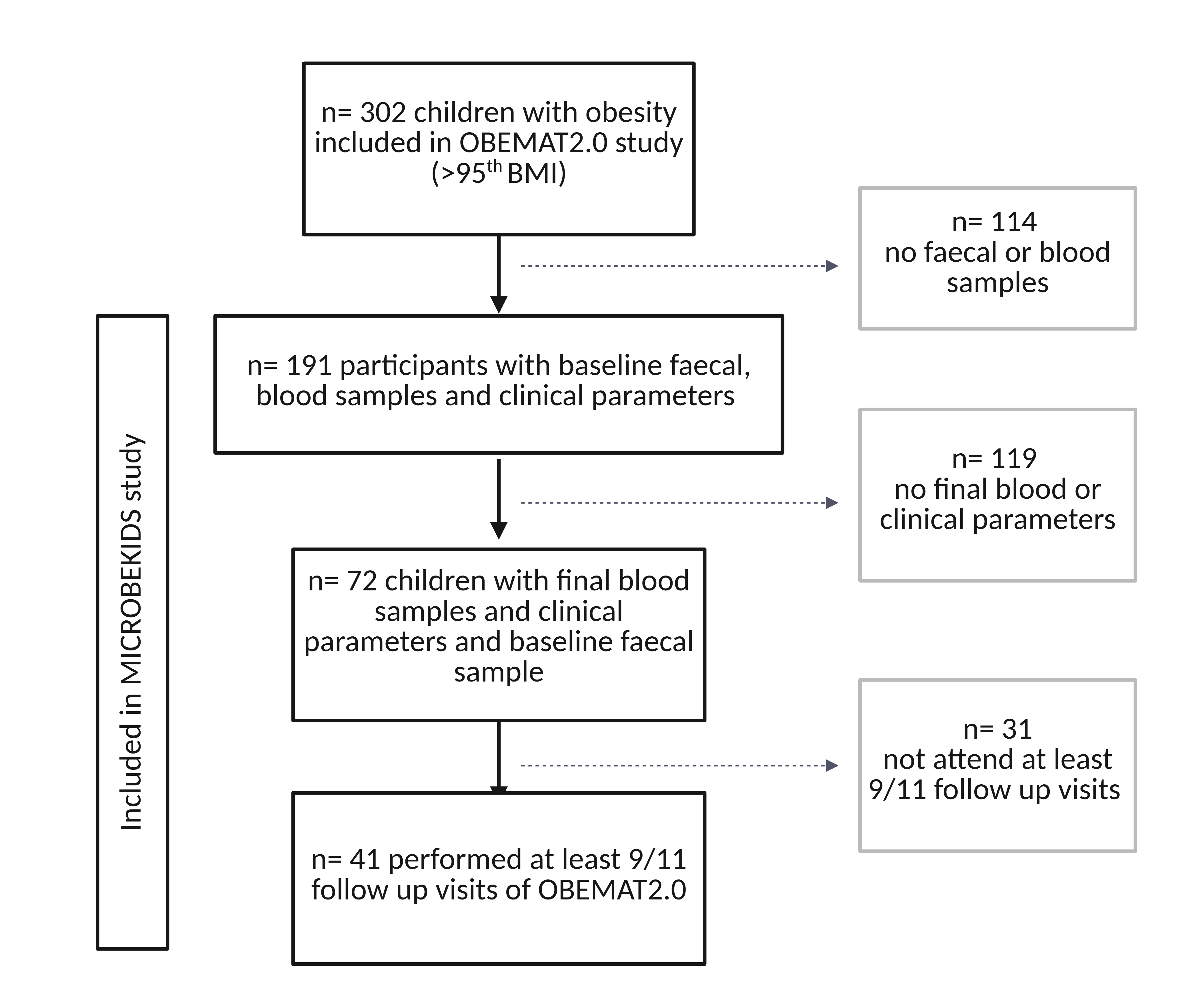

Supplement: AlcazarM_GutMicrobiota_FigureS1.png — Supplemental Material [file KGMI_A_2631824_SM3083.png]
