# Supplementary material for: Children’s gut microbiota predicts the efficacy of obesity treatment
Source: Gut Microbes. 2026 Feb 19;18(1):2631824. doi: 10.1080/19490976.2026.2631824 (PMC12928635; doi:10.1080/19490976.2026.2631824)
Supplement: Supplementary_figures.docx — Supplemental Material [file KGMI_A_2631824_SM3081.docx]

**Supplementary Figure 1.** Flowchart of the participants included in the study

**Supplementary Figure 2.** Abundances of top-enriched bacteria in response groups. These plots compare the relative abundances of the significant bacteria obtained in the LEfSe analysis in both MetScore (A) and BMI z-score (B) , respectively. s_: unknown species of the reported genus. C.group: *coprostanoligenes* group.
